# Oxovanadium(IV) Coordination Compounds with Kojic Acid Derivatives in Aqueous Solution

**DOI:** 10.3390/molecules24203768

**Published:** 2019-10-19

**Authors:** Silvia Berto, Eugenio Alladio, Pier Giuseppe Daniele, Enzo Laurenti, Andrea Bono, Carmelo Sgarlata, Gabriele Valora, Rosita Cappai, Joanna Izabela Lachowicz, Valeria Marina Nurchi

**Affiliations:** 1 Dipartimento di Chimica, Università di Torino, Via Pietro Giuria 7, 10125 Torino, Italy; eugenio.alladio@unito.it (E.A.); piergiuseppe.daniele@unito.it (P.G.D.); enzo.laurenti@unito.it (E.L.);; 2 Dipartimento di Scienze Chimiche, Università degli Studi di Catania, Viale Andrea Doria 6, 95125 Catania, Italy; sgarlata@unict.it (C.S.); gabriele.valora@unict.it (G.V.); 3 Dipartimento di Scienze della Vita e dell’Ambiente, Università di Cagliari, Cittadella Universitaria, 09042 Cagliari, Italy; cappai@unica.it (R.C.); nurchi@unica.it (V.M.N.); 4 Dipartimento di Scienze Mediche e Sanità Pubblica, Università di Cagliari, Cittadella Universitaria, 09042 Monserrato, Italy; lachowicz@unica.it

**Keywords:** vanadium, kojic acid, UV-visible spectroscopy, EPR spectroscopy, potentiometry, chemometry

## Abstract

Hydroxypyrone derivatives have a good bioavailability in rats and mice and have been used in drug development. Moreover, they show chelating properties towards vanadyl cation that could be used in insulin-mimetic compound development. In this work, the formation of coordination compounds of oxovanadium(IV) with four kojic acid (5-hydroxy-2-(hydroxymethyl)-4-pyrone) derivatives was studied. The synthetized studied ligands (S2, S3, S4, and SC) have two or three kojic acid units linked through diamines or tris(2-aminoethyl)amine chains, respectively. The chemical systems were studied by potentiometry (25 °C, ionic strength 0.1 mol L^−1^ with KCl), and UV-visible and EPR spectroscopy. The experimental data were analyzed by a thermodynamic and a chemometric (Multivariate Curve Resolution–Alternating Least Squares) approach. Chemical coordination models were proposed, together with the species formation constants and the pure estimated UV-vis and EPR spectra. In all systems, the coordination of the oxovanadium(IV) starts already under acidic conditions (the cation is totally bound at pH higher than 3–4) and the metal species remain stable even at pH 8. Ligands S3, S4, and SC form three coordination species. Two of them are probably due to the successive insertion of the kojate units in the coordination shell, whereas the third is most likely a hydrolytic species.

## 1. Introduction

**Figure 1:**
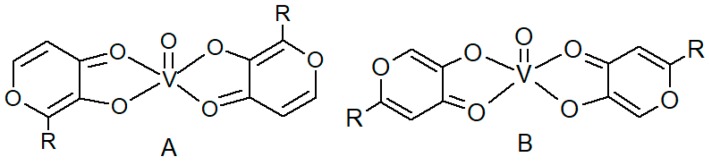
Structures of hydroxy-pyronato-oxovanadium(IV) coordination compounds. (**A**) Structure of bis(2-alkyl-3-hydroxy-4-pyronato)oxovanadium(IV); (**B**) structure of bis(2-alkyl-5-hydroxy-4-pyronato)oxovanadium(IV).

Vanadium plays different roles in biological systems [1] and shows a variety of pharmacological properties in humans [2,3], even if it is not an essential metal ion [4]. In particular, vanadyl salts and vanadyl coordination compounds exhibit insulin-mimetic activity and may be good candidates for the treatment of type II diabetes mellitus [3]. Bis(maltolato)oxovanadium(IV) (BMOV) and bis(ethylmaltolato)oxovanadium(IV) (BEOV) have undergone extensive pre-clinical testing, and BEOV has been promoted to Phase II clinical trials [5,6]. These coordination compounds show a peculiar structure where two 3-hydroxy-4-pyrone units are linked in the equatorial plane of the vanadyl cation coordination sphere, as shown in Figure 1A [7].

Hydroxypyrones are present in plant products, and many of their derivatives exhibit low toxicity, therefore good biocompatibility, and have been used for drug development [8]. Kojic acid (5-hydroxy-2-(hydroxymethyl)-4-pyrone, ka) is a fungal metabolite. ka and its derivatives are promising chelating agents for vanadyl cation and could be used in insulin-mimetic drug development. The coordination of ka or its derivatives with oxovanadium(IV) was previously studied. Yuen et al. [9] compared the glucose-lowering properties of BMOV and bis(kojiate)oxovanadium(IV) species, showing that the latter is effective but the BMOV is more potent. Sanna et al. [2] studied the chemical equilibria of ka–vanadyl species in blood serum and showed that the behavior of ka reflects that of maltol. Wei et al. [10,11] developed and tested a series of coordination compounds based on the ka structure for glucose control in blood, which showed similar glucose-lowering activity and lower toxicity with respect to BMOV.

**Figure 2:**
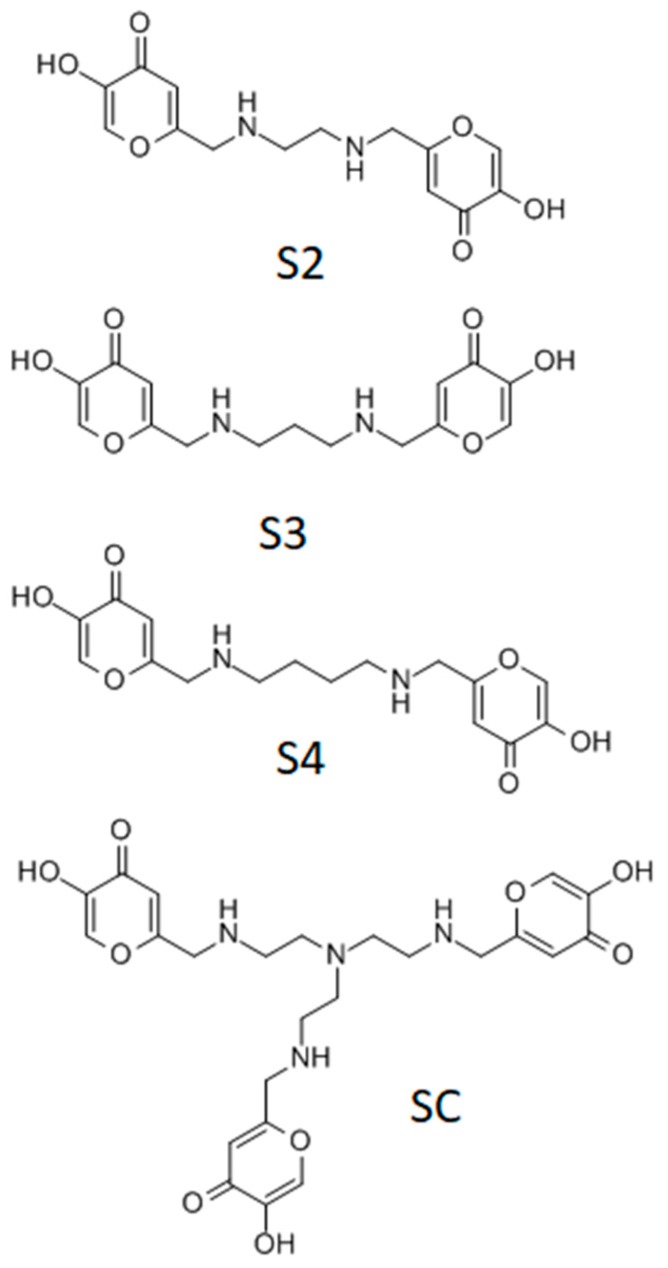
Molecular structure of the ka derivatives studied. S2: [ethane-1,2-diylbis(iminomethanediyl)]bis(5-hydroxy-4H-pyran-4-one), S3: [propane-1,3-diylbis(iminomethanediyl)]bis(5-hydroxy-4H-pyran-4-one); S4: [butane-1,4-diylbis(iminomethanediyl)]bis(5-hydroxy-4H-pyran-4-one); SC: 6,6′,6″-(((nitrilotris(ethane-2,1-diyl))tris(azanediyl))tris(methylene))tris(3-hydroxy-4H-pyran-4-one).

In the present work, a series of ka-based ligands were employed for the coordination of the vanadyl cation. The synthesis and the characterization of the four investigated ligands were previously reported, as well as their coordination capability towards Fe^3+^, Al^3+^, Cu^2+^, and Zn^2+^ cations [12,13]. The ligand structure presents two or three ka units linked through ethylene diamine, propylene diamine, butylene diamine, and tris(2-aminoethyl)amine, as shown in Figure 2. These molecules can be easily synthetized at high purity level and can interact with different metal cations through alcoholate, carbonyl, and amino groups. The presence of different donor groups gives the ligands a strong chelating ability towards both hard and soft metal cations. Moreover, the flexibility of the ligand chain permits the formation of 1:1 (metal:ligand) stoichiometry species [12]. The alkyl chains, which do not coordinate metal ions, could increase the lipophilicity of the molecules and, therefore, ameliorate the pharmacological effectiveness [10].

The coordination features of ligands S2–S4 and SC with oxovanadium(IV) were studied by potentiometry, and UV-visible and EPR spectroscopy. Chemical models were hypothesized on the basis of experimental data, which were analyzed by a thermodynamic approach and chemometric methods. The spectroscopic data, in particular, were analyzed by HypSpec^®^ software [14] and by the Multivariate Curve Resolution–Alternating Least Squares (MCR–ALS) method [15]. The formation constants of the species and the pure UV-vis and EPR spectra were determined.

## 2. Results and Discussion

### 2.1. Potentiometric Data

pH-metric titrations were carried out in water solutions with different metal to ligand ratios (ionic strength 0.1 molL^−1^ with KCl, 25 °C) in the 2–11 pH range. As an example, some titration curves are shown in Figures S1–S8 in the Supplementary Material file. The curves obtained on all systems in the presence of the cation showed a marked acidification of the solution due to the formation of the coordination compounds (Figures S1–S4). The first equivalent point is correlated with the excess of H^+^ in the solutions and with the protons released during the cation coordination. For all systems, it was possible observe the release of two protons for each ligand molecule (Figures S1–S4), except for S2. The solutions are yellow–green at acidic pH and yellow in alkaline conditions. The equilibrium becomes unachievable after the equivalent point for the solutions with a metal to ligand ratio of 1:1, except for the SC ligand, while instability starts above pH 8.5 with ligand excess. In any case, when instability occurs, the solutions acquire a dark-orange color. The typical browning of the solutions reveals that the vanadyl cation is being involved in reactions different from cation coordination (precipitation and/or oxidation of vanadium(IV) to vanadium(V)) [16]. Back titrations were conducted on each system, proving the reversibility of the equilibria in the pH range 2.0–8.0 (Figures S5–S8). For this reasons, the titrations were carried out preferentially in an excess of the ligand, and only the titration data collected between pH 2.0 and 8.0 were processed by the BSTAC program [17]. The chemical models proposed, with the formation constants of the species, are listed in Table 1.

The ligand S2 showed the formation of a main species, [VOLH_3_]^3+^, before the equivalent point and of a second one, [VOLH_2_]^2+^, above pH 5.

The titration curves obtained with S3 and S4 can be explained by the formation of two species, [VOLH_3_]^3+^ and [VOLH_2_]^2+^, in which the hydroxyl group of each ka units probably dissociate successively at a very lower pH with respect to the free ligand molecules [12,13]. Both species are formed before the equivalent point. The buffer region of the titration curves could be explained on the basis of the ligand deprotonation only, but the trends of the UV-vis spectra recorded on the systems suggest the formation of a third species after pH 6.5 (see the next paragraph). Therefore, the species [VOLH]^+^ was also included in the model. Their percentage formation is lower than 10% in the pH range considered; therefore, they can be considered as minor species and their formation constants are affected by larger uncertainties.

Regarding SC ligand, the formation of three different species, [VOLH_5_]^+4^, [VOLH_4_]^+3^, and [VOLH_3_]^+2^, was assumed. Both [VOLH_5_]^+4^ and [VOLH_4_]^+3^ are formed before the equivalent point.

The models proposed well explain the experimental titration curves (see Figures S5–S8); nevertheless, the uncertainty of the stability constants results to be quite high because their values are strictly correlated to each other, and the species start to be formed in quite acidic conditions where the variation of the proton concentration due to the formation of the coordination compounds is a small fraction of the total proton concentration in solution. The presence of dimeric species was tested, but their formation was excluded. On the other hand, if a stable dimeric species had been formed, the stability of solutions with a metal to ligand ratio of 1:1 would have been higher.

The general notation (VO)_p_L_q_H_r_^2p+r−qz^ used to define the coordination compounds does not allow differentiating a proton resulting from the dissociation of the ligand from a proton deriving from a water molecule, but it is possible to hypothesize on the basis of the partial formation constants obtained on the different metal–ligand systems. For all systems, it is reasonable assume that the species [VOLH_3_]^+3^ of S2–S4, and the species [VOLH_5_]^+4^ of SC, are due to the insertion of one ka unit in the coordination sphere of the cation that bind the metal through the carbonyl oxygen atom and the phenolic group, (CO, O^−^). Buglyó et al. [18] and Yuen et al. [9] reported formation constants of ~7.6 for the species [VOka]^+^ (Table 1).

From the log*K* of the species [VOLH_2_]^+2^ (S3–S4) and [VOLH_4_]^+3^ (SC), it is reasonable assume the coordination of the cation through two ka units, 2 × (CO, O^−^), while, in the case of S2, it is more probable the formation of a hydrolytic species with the stoichiometry [VO(H_3_S2)(OH)]^+2^ because the log*K* value is very low with respect to those estimated for the other ligands. On the other hand, S2 is the ligand with the shortest amine chain, and this may hinder the coordination by the second (CO, O^−^) group. Moreover, S2 is the ligand with the highest proton dissociation constants of the ka units (Table 1).

Oxovanadium(IV) species with stoichiometry ML formed by the insertion of two units of 3-hydroxy-6-methyl-4-pyrone linked through an ethylenediamine chain were previously observed by Song et al. [19] for the ligand *N,N’*-bis(3-hydroxy-6-methyl-2-methylene-4-pyrone)ethylenediamine.

The third species of S3, S4, and SC ([VOLH]^+^ or [VOLH_3_]^+2^) is probably a hydrolytic species with stoichiometry, [VO(H_2_S3)(OH)]^+^, [VO(H_2_S4)(OH)]^+^, and [VO(H_4_SC)(OH)]^+2^, respectively. In the cases of S3 and S4, the proton of the water molecule shows a dissociation constant quite similar to those of the protogenic groups of the ligands; therefore, on the basis of potentiometric data only, it is tricky to detect these species.

**Figure 3:**
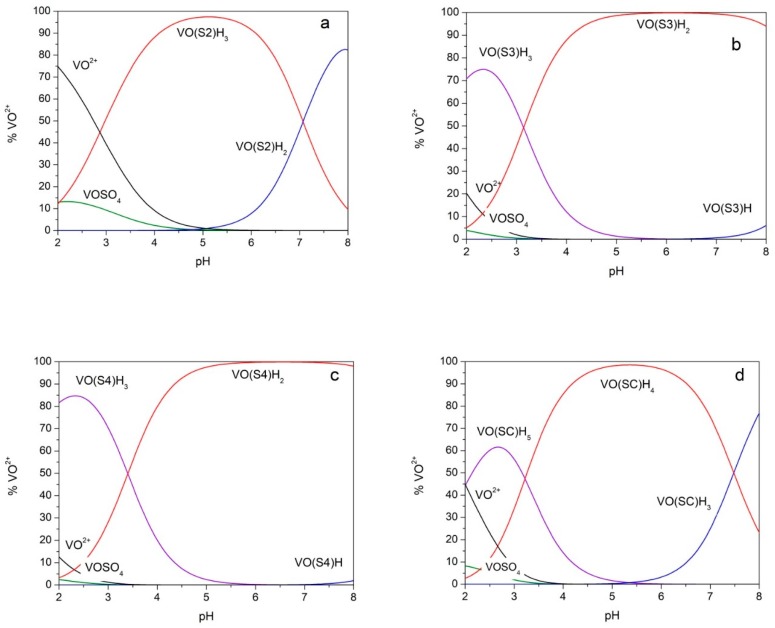
Species distribution diagrams. Species distribution diagrams for solutions of oxovanadium(IV) 5 mmolL^−1^ and ligand 10 mmol L^−1^ calculated on the basis of the formation constants reported in Table 1. (**a**) ligand = S2; (**b**) ligand = S3; (**c**) ligand = S4; (**d**) ligand = SC. VOSO_4_: Vanadyl–sulphate ion pair.

The species distribution diagrams of the four studied systems were calculated on the basis of the data in Table 1 and are shown in Figure 3. It can be observed that, for systems with S3, S4 and SC, above pH 4, the species that involves two (CO, O^−^) donor sets is predominant.

### 2.2. UV-Visible Spectra

**Figure 4:**
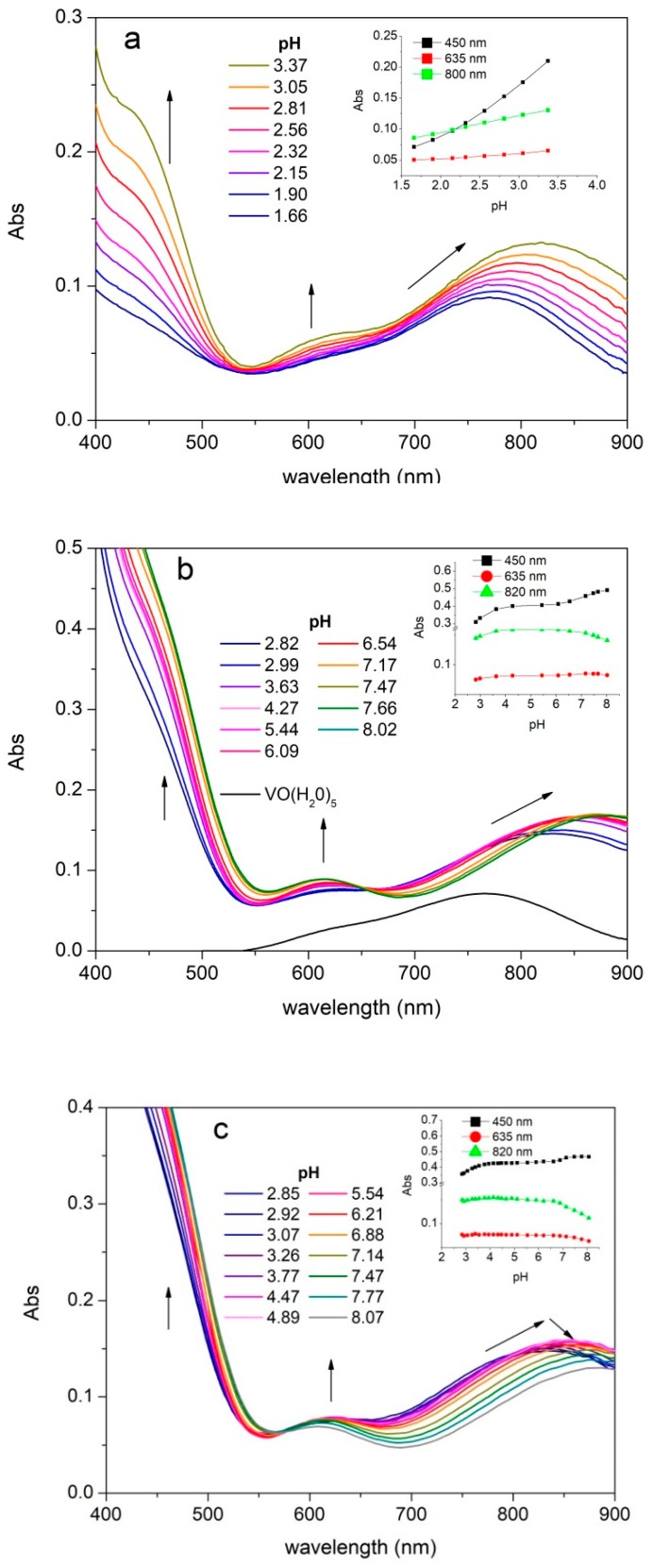
UV-vis absorption spectra. UV-vis absorption spectra of a solution containing oxovanadium(IV) 5 mmolL^−1^ and S2 5 mmolL^−1^ (batch titration) (**a**), S3 15 mmol L^−1^ (**b**), and SC 15 mmol L^−1^ (**c**). Inset graphs: Diagrams Abs vs. pH at different wavelengths.

UV-vis absorption spectra were recorded on solutions of vanadyl cation and the different ligands as a function of pH. The experimental spectra of all studied systems show three absorption bands between 400 and 900 nm due to the cation species (namely band *I*, *II,* and *III* from the highest to the lowest wavelength). As shown in Figure 4, that reports the experimental spectra recorded on the systems with S2, S3, and SC, the λ_max_ values of the band *I* are between 820 and 890 nm. The band *I* is positioned at 765 nm for the pentaaquo ion [VO(H_2_O)_5_]^2+^, therefore the chelation due to the (CO, O^−^) donors provides a strong red shift, which is evident already at acidic pH (Figure 4a and Figures S9–S11). The second absorption band (band *II*) is positioned between 625 and 590 nm, whereas the corresponding λ_max_ of the pentaaquo ion is at 635 nm. The third band (band *III*) is visible at about 455 nm as a shoulder of the ligand absorption band tail. The coordination leads to a hyperchromic effect for all the three transitions. The systems showed stable potentials until pH 8. All systems showed a stability of the absorption values from pH 4.5 to 6.5 followed by a regular decrease in absorbance at higher pH associated with a further bathochromic shift of the band *III* (Figure 4c).

For each investigated system, from the whole of the spectra recorded on solutions at different concentrations and pH, the individual visible spectra were determined by the analysis of experimental data (wavelengths 550–900 nm) with HypSpec^®^. The principal spectral features of each species are listed in Table 2, and the entire spectra and the formation constants estimated are reported in the Supplementary Material file (Figures S12–S15 and Table S1). The percentage formation of the species [VOLH]^+^ of S3 and S4 are lower than 10% in the pH range considered; therefore, the spectral parameters should be considered semi-quantitative. The position of the absorption maxima can be associated with the species proposed on the basis of the potentiometric data (see the donor set in Table 2). The entrance of one (CO, O^−^) group in the coordination sphere moves the band *I* from 765 to 817–851 nm, whereas the metal chelation through two (CO, O^−^) groups shifts the absorption maxima to the range 854–861 nm. The hydrolyzed species show absorption maxima in the range 884–898 nm. For the species due to the coordination of one (CO, O^−^) group, it is not possible exclude the participation of a second ka unit through a CO group.

The position of the metal species bands is in quite good accordance with the absorption maxima obtained by Buglyó et al. [18] for the ka-oxovanadium(IV) species, and with those obtained by Chruscinska et al. [20] for the coordination compounds of the cation with maltol and L-mimosine (see Table 2). In the case of L-mimosine (α-amino-β-(3-hydroxy-4-oxo-1,4-dihydropyridin-1-yl)-propanoic acid), the participation of the amino groups in coordination was excluded [20]. These findings support the hypothesis that only the oxygenated functions of the ligands participate to the oxocation coordination.

### 2.3. EPR Spectra

EPR spectra of the different vanadyl–ligand systems were recorded at both room (RT) and low (LT) temperature.

**Figure 5:**
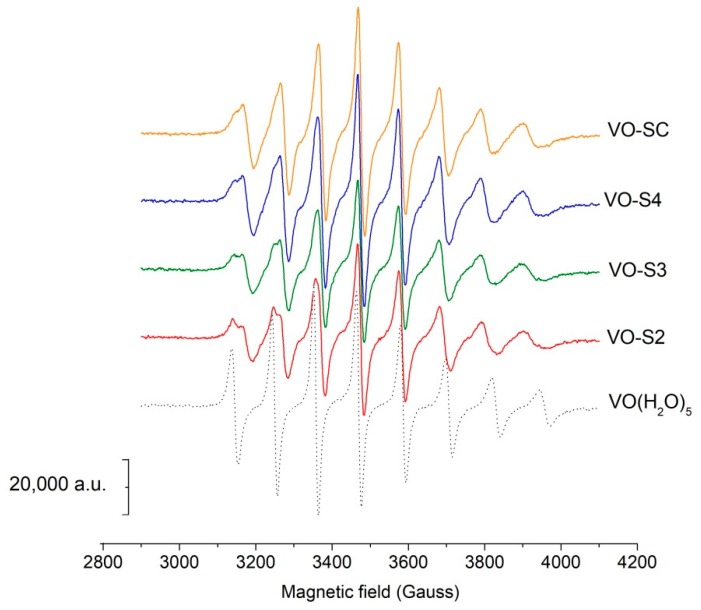
RT EPR spectra in acidic conditions. EPR spectra of solutions with oxovanadium(IV) 5 mmol L^−1^ and different ligands 15 mmol L^−1^ at pH 2.5.

The RT EPR spectra showed the typical eight line pattern of vanadyl mononuclear species, due to the coupling of the unpaired electron with ^51^V nucleus (*S* = 1/2, *I* = 7/2). With increasing pH, the values of the isotropic hyperfine coupling constant *A*_0_ decreased, revealing the progressive substitution of the water molecules in the pentaaquo ion [VO(H_2_O)_5_]^2+^ with one or more ligand donor groups. Some of the experimental spectra obtained are reported in Figure 5 and Figure S16. At acidic pH (Figure 5), it is possible to note the presence of two partially overlapped signals that can be reasonably attributed to the pentaaquo ion and to the species [VOLH_3_]^+3^ (L = S2, S3 or S4) or [VO(SC)H_5_]^+4^. Increasing the pH only one signal can be observed. The experimental data were simulated with the program SIM32 [22,23] and deconvoluted by MCR–ALS as well [24]. The values of *A_0_* and *g_0_*, derived from both the data treatments, are shown in Table 3.

The MCR–ALS approach provided good results. An example is reported in Figure S17 (in the Supplementary Material file) that shows extrapolated pure concentration profiles and spectra of a series of solutions involving oxovanadium(IV) 5 mmol L^−1^ and S3 15 mmol L^−1^ that were prepared at different pH values. MCR–ALS decompositions were performed by using the EPR spectrum of the pure oxovanadium(IV) as reference. Two- and three-component models were calculated and compared in terms of explained variance and lack of fit values. In all cases, the systems involving a three-component solution provided better results than those with two estimated components, showing satisfactory lack of fit and explaining variance values lower than 10% and higher than 99%, respectively. Moreover, a comparison between the pure EPR spectra of the standard of oxovanadium(IV) with the profile extracted by MCR–ALS (see Figure S18 reported in the Supplementary Material file) showed an almost complete overlap between the two spectra. The *A*_0_ and *g*_0_ values (Table 3) obtained by MCR–ALS were quite similar to those obtained from SIM32. Both SIM32 and MCR–ALS did not allow distinguishing between the signals due to species formed in alkaline conditions, but the decrease of the *A_0_* values with increasing pH suggests its presence.

**Figure 6:**
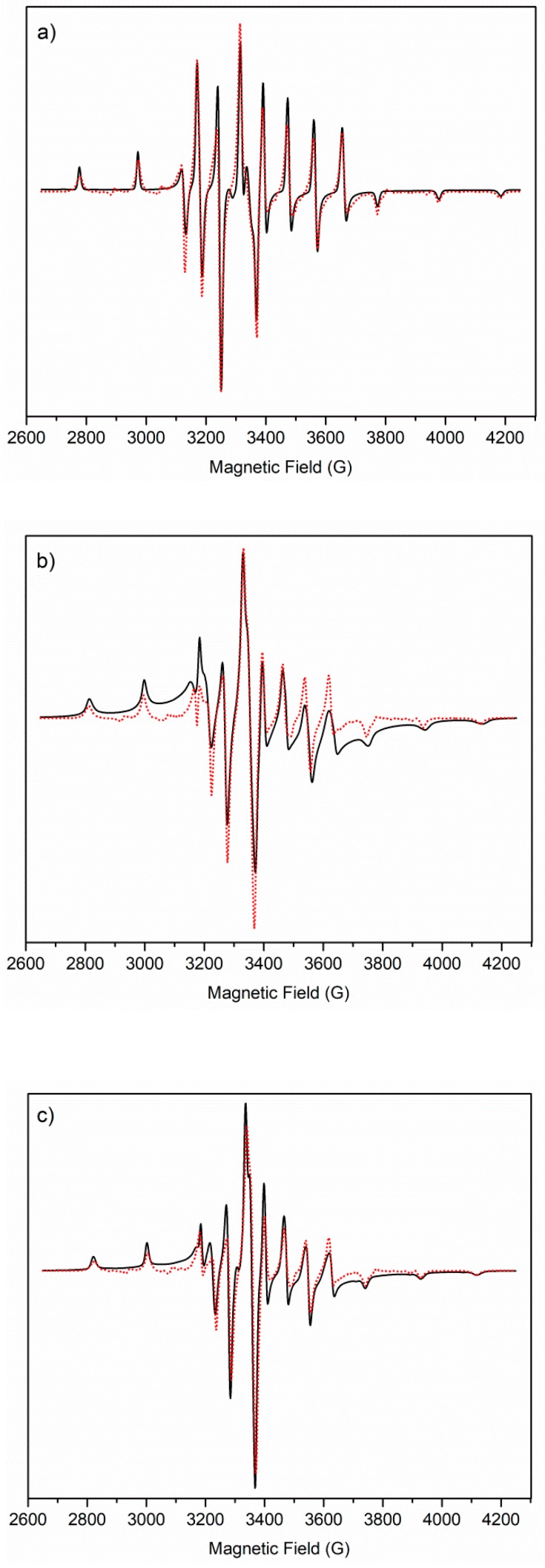
LT EPR spectra. Experimental (black solid line) and computed (red dot line) frozen solution EPR spectra of (**a**) VO^2+^ pentaaquo ion, (**b**) VO–SC at pH 5.6, and (**c**) VO–SC at pH 8.

LT EPR spectra were recorded on frozen VO^2+^–ligand solutions at pH 6 and 8. The data show only little differences. According to the RT EPR spectra of the vanadyl compounds with SC ligand, a multispecies LT EPR spectrum is obtained at pH around 3 (see Figure S19 in the Supplementary Material file). Remarkably, low temperatures might change the species distribution determined at room temperature, as in the case of copper(II) complexes, generally favoring metal species with a higher coordination level [25]. Table 4 reports anisotropic magnetic parameters at different pH values for all the vanadyl species with ka derivatives, as well as those for the pentaaquo oxovanadium(IV) ion. Figure 6 shows, as an example, the LT frozen solution EPR spectra of the oxovanadium(IV) coordination compounds with the SC ligand at different pH values.

From inspection of the Table 4, it is quite evident that the ligands S3, S4, and SC replace equatorial water molecules of the pentaaquo oxovanadium cation with both the bidentate oxygen moieties of the ligands (CO, O^−^). The shifts on *g* and *A* values are those expected in the case of bidentate ligands, as those observed for β-diketonates, in which oxygen atoms are more covalently bound than water molecules, and therefore higher *g*_‖_ and lower *A*_‖_ values are expected [26,27,28]. The *A*_‖_ values approximately obey to the additivity relationship for equatorial ligands, as explained by Smith et al. [26], confirming the presence of four equatorial oxygen donors. Moreover, the values reported in Table 4 are in very good agreement with those proposed by Buglyò at al. [18] for the species VO(ka)_2_ (*g*_‖_ = 1.939; *A*_‖_ = 171 × 10^−4^ cm^−1^).

It was not possible to differentiate the signals of hydrolytic species, but little increase of the g_‖_ values and decrease of A_‖_ values can be observed for the data collected at pH 8, suggesting their presence. Moreover, the results obtained for S2 show little difference with respect to those obtained for other systems, despite potentiometric results suggesting the insertion of only one (CO, O^−^) donor set. This could be due to the similar contribution of the hydrolytic species signal to the resultant A_‖_ and g_‖_ parameters.

## 3. Sequestering Capability

In order to define the sequestering ability of the ligands under study towards oxovanaium(IV) and compare it with those of ka and maltol, the sum of formation percentages of all metal–ligand species (Σ%VO^2+^_compl_) was plotted versus the pL, where pL = −log_10_[L]_tot_ ([L]_tot_ is the total ligand concentration) for each metal–ligand system. This procedure was proposed by Crea et al. [29] and provides curves that show an exponential decay. The higher the pL necessary to bind a defined percentage of metal, the better the sequestering capability.

**Figure 7:**
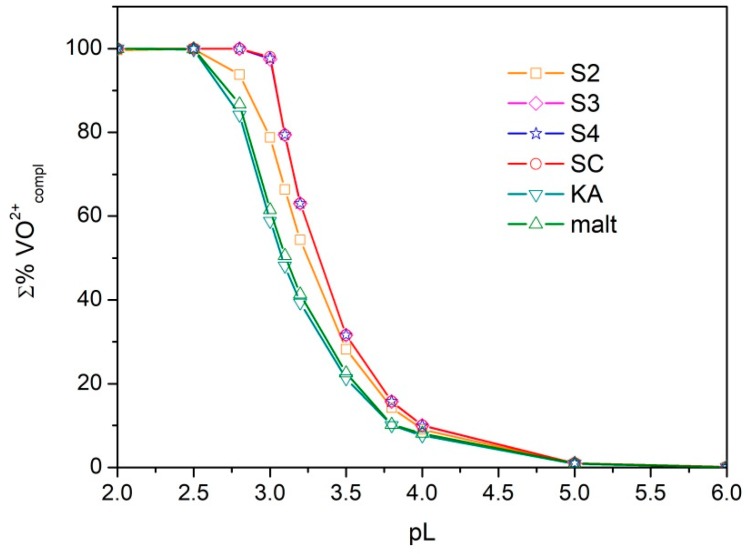
Sequestration diagram. Sum of formation percentages of all metal–ligand species vs. pL values, where L = S2–S4, SC, ka, or maltol. Experimental conditions: I = 0.1 mol L^−1^ in KCl, 25 °C, pH = 5.5.

The obtained curves are shown in Figure 7. Σ%VO^2+^_compl_ was calculated at pH 5.5 and I = 0.1 molL^−1^. At this pH value, the ligands exhibit their maximum binding capacity. For ka and maltol, the chemical models proposed by Buglyó et al. [18] were used and the formation constants were extrapolated at ionic strength 0.1 molL^−1^ using an expanded Debye–Hückel equation [30]. The sequestering ability of the ligands S3, S4, and SC are very similar and their curves are overlaid; moreover, they show higher sequestering capability with respect to ka or maltol.

## 4. Materials and Methods

### 4.1. Chemicals

The stock solution (~0.1 mol L^−1^) of vanadyl sulphate (purity ~96%, Aldrich, St. Louis, MO, USA) was weekly prepared without previous purification of the salt; it was standardized by redox titration with permanganate solution (Carlo Erba, Milan, Italy), followed by photometric detection [21]. D_2_O (99.9%), ethanol (99.9%), 5-hydroxy-2-hydroxymethyl-pyran-4-one (kojic acid, ka; purity 99%), ethylene diamine, propane-1,3-diamine, and butane-1,4-diamine were Sigma-Aldrich products. Ethane-1,2-diylbis(iminomethanediyl)]bis(5-hydroxy-4H-pyran-4-one), propane-1,3-diylbis(iminomethanediyl)]bis(5-hydroxy-4H-pyran-4-one), butane-1,4-diylbis(iminomethanediyl)]bis(5-hydroxy-4H-pyran-4-one), and 6,6′,6″-(((nitrilotris(ethane-2,1-diyl))tris(azanediyl))tris(methylene))tris(3-hydroxy-4H-pyran-4-one) (hereinafter S2, S3, S4, and SC, respectively) were synthetized as reported in references. [12,13]. The identity and the purity of the synthetized ligands were evaluated by NMR spectroscopy (Bruker Ascend™ 400 MHz spectrometer; Bruker Italia, Milan, Italy) and the protonation level was checked by alkalimetric titration.

Potassium chloride solutions were prepared by weighing the pure salt (Fluka, p.a., Seelze, Germany). Standard KOH (0.1 and 0.5 mol L^−1^) and HCl solutions (1.0 and 0.1 mol L^−1^) were prepared by diluting Merck (Darmstadt, Germany) concentrate products, and standardized against potassium hydrogenphthalate (Fluka, purity ≥99.5%) and sodium carbonate anhydrous (Fluka, purity 99.95–100.05%), respectively. All solutions were prepared using grade A glassware and ultrapure water (conductivity <0.1 μS).

### 4.2. Potentiometric Measurements

Potentiometric measurements were performed using the Metrohm (Herisau, Svizzera) automated titration system Titrando 888 controlled by the software *tiamo*^TM^ 2.5, or a Metrohm mod. 713 potentiometer (resolution of ±0.1 mV) coupled with a Metrohm 765 Dosimat burette (minimum volume deliverable of ±0.001 cm^3^). Metrohm combined glass electrodes (mod. 6.0259.100 and mod. 6.0234.100) with internal reference Ag/AgCl/3M KCl were used. The potentiometric titrations were carried out in a stream of purified nitrogen gently bubbled in the titration cell to avoid O_2_ and CO_2_ contamination. The measurement cells were thermostated at (25.0 ± 0.1 °C) by means of water circulation from a thermocryostat (mod. D1-G Haake, Vreden, Deutschland). A maximum signal drift of 0.5 mVolt min^−1^ and a maximum waiting time of 100 s were imposed for each titration point. The Dynamic Titration mode was used, the minimum titrant increment was 0.01 mL, and the measuring point density was set in order to have ΔE < 15 mV for each titrant addition. The titration curves obtained by the automated titration system on each chemical system were compared to those recorded manually in order to verify the equilibrium condition. Some back titrations were conducted (titrant HCl 0.1 mol L^−1^) on each system in order to check the reversibility of the equilibria.

The electrode couple was standardized, in terms of pH = −log[H^+^], by titrating HCl 10 mmol L^−1^ solution (at the same ionic strength value as the solution under study) with standard KOH, in order to determine the formal potential *E*^0^ before each experiment.

The potentiometric titrations were carried out in KCl aqueous solutions with 0.1 mol L^−1^ ionic strength. For the investigation of VO^2+^–ligand systems, 25 or 50 mL of solution containing VO^2+^, the ligand, and KCl were titrated with standard KOH. Each titration was repeated at least twice. The metal concentration ranged from 2 to 10 mmol L^−1^. The ligand concentration ranged from 2 to 15 mmol L^−1^. The metal to ligand ratios were 1:1, 1:2, 1:3, 2:1, and 3:1. The investigated pH range was 2.0–11.0 and, since the synthetized ligands were not completely protonated, the initial acidic condition was obtained by the addition of HCl 0.1 mol L^−1^.

Before use, the ligands (S2–S4, SC) were kept at 60 °C for a night. This treatment enabled to remove most of the solvent derived from the synthetic procedure, and therefore, it allowed obtaining a constant weight.

### 4.3. Spectroscopic Measurements

The visible molecular absorption (400–900 nm) spectra were recorded by a V-550 Jasco double-beam spectrophotometer (JASCO Europe, Cremella, Italy), equipped with Hellma quartz cuvettes (1.000 cm optical path length, sample in a cell for flow-through measurements). The signal was collected each 1 nm, with a scan rate of 400 nm min^−1^, and the baseline was taken in air before each absorbance measurement. Each absorbance spectrum was taken against the reference cuvette filled with KCl 0.1 mol L^−1^. The solution being examined was transferred from the potentiometric to an optical cell using a peristaltic pump. Due to the low values of the molar absorptivity coefficients of the vanadyl-containing species, the concentration of oxocation was always higher than 5 mmol L^−1^, with the suitable metal to ligand ratio. In order to better estimate the formation constants of the species, some batch titrations were conducted at very acidic conditions in a mixture of HCl and KCl, maintaining constant the ionic strength at 0.1 mol L^−1^.

The EPR spectra of VO^2+^-ligand systems were recorded at room temperature (RT) with an ESP-300E Bruker X-band spectrometer (Bruker Italia, Milan, Italy). Experimental parameters were as follows: Number of scans 5; microwave power 2 mW; microwave frequency 9.78 GHz; modulation amplitude 0.4 mT; modulation frequency 100 KHz; time constant 82 ms; sweep time 84 s.

Low temperature (LT) EPR spectra of oxovanadium(IV) frozen solutions were recorded by a Bruker Elexsys E500 CW-EPR spectrometer (Bruker Italia, Milan, Italy) driven by a PC running XEpr program under Linux, equipped with a Super-X microwave bridge operating at 9.3–9.9 GHz and a SHQE cavity. Instrumental settings of frozen solution EPR spectra were as follows: Number of scans 1–3; microwave frequency 9.42–9.45 GHz; modulation frequency 100 kHz; modulation amplitude 1 mT; time constant 164–327 ms; sweep time 6 min; microwave power 10–20 mW; linear receiver gain 1 × 10^4^ to 1 × 10^5^. All the EPR frozen solution spectra were recorded in quartz tubes at 150 K by means of a ER4131VT variable temperature apparatus.

The EPR spectra were recorded on solutions with a metal to ligand ratio of 1:3. The RT spectra were recorded on solutions with vanadyl 5 mmol L^−1^, whereas the LT EPR spectra were recorded on solutions with vanadyl 1–4 mmol L^−1^. The ionic strength was maintained at 0.1 mol L^−1^ with KCl and the pH of the solutions was comprised between 2.5 and 8.0. Methanol up to 5% was added to the aqueous solution in order to increase the resolution of LT frozen solution spectra.

### 4.4. Data Analysis and Calculations

The electrode calibration data were analyzed by the ESAB2M program [31] in order to refine the electrode parameters. This program was used to refine the formal potential *E*^0^, the Nernstian slope, and the analytical concentration of the reagents.

The refinement of the formation constants was performed by the BSTAC software [17]. It employs an iterative and convergent numerical method, which is based upon the linear combination of the mass balance equations, minimizes the error squares sum on electromotive force values. The data refinement was performed by including in the model the protonation constants of the ligands, reported in previous papers [12,13] (see Table 1), the hydrolysis constants of the vanadyl cation ([VO(OH)]^+^ logβ = −5.65; [(VO)_2_(OH)_2_]^+2^ logβ = −7.02), and the formation constant of the vanadyl–sulphate ion pair ([VO(SO_4_)]^0^ logβ = 1.73) [21]. The p*K*_w_ was 13.78 and both total ligand and proton concentration were refined.

Spectrophotometric data were analyzed by means of the HypSpec^®^ program [14] that optimizes the formation constants and the values of molar absorptivity coefficients (ε_λ_/L mol^−1^ cm^−1^) of the different species applying mass balance equations and Lambert–Beer’s law. The experimental spectra (absorbance vs. wavelength λ/nm), the analytical concentrations of the reagents, and the proposed chemical model (stoichiometric coefficients and known stability constant values of all species) were the input data. The absorbance values comprised between 550 and 900 nm were considered in order to exclude from the data treatment the ligand absorption. The [VO(SO_4_)]^0^ was considered as an absorbing species during the elaboration process and the spectra obtained were compared with that presented in a previous paper [16].

RT EPR spectra of VO^+2^ solutions showed typical unequal spacing between the lines due to second order effects. Therefore, in order to obtain the correct values of *g* and *A*, experimental data were analyzed with the EPR simulation program SIM32, written by Spałek and Sojka [22,23].

Anisotropic magnetic parameters of the LT EPR spectra were obtained by simulating the experimental spectra through a modified program from Pilbrow et al. [32], in which the line width (LW) parameters are allowed to vary according to the general formula LW = LW ± *a*M_i_*^b^*, where *a* is a multiplication coefficient and *b* is an exponent of the nuclear magnetic component of the vanadium nucleus multiplet.

UV-vis and EPR spectra were computed by Multivariate Curve Resolution–Alternating Least Squares (MCR–ALS). MCR is a chemometric strategy that performs a bilinear decomposition of the original data set X, consisting of N spectra/samples, into the product of two distinct matrixes, C and S, plus the residual error E [33,34,35,36]. In particular, S is referred to contain a number of n pure spectra (where n < N) relative to the specific constituents that have been extracted from the samples under investigation. On the other hand, C includes the information about the signal-related concentration profiles of the pure aforementioned constituents of the samples. The general MCR procedure is summarized by the following formula: X = C S^T^ + E. At the same time, the altering least squares (ALS) approach is performed in order to minimize the deconvolution error by involving specific constraints (such as, for instance, unimodality, non-negativity, equality, and concentration closure) [35,36,37] that allow to optimize the estimation of the pure spectra and the concentration profiles. In more detail, the whole MCR–ALS procedure starts with the estimation of the number of compounds (components) that constitute the examined samples, and this is performed by involving Principal Component Analysis (PCA) or Single Value Decomposition (SVD) protocols [36,38]. Then, a preliminary bilinear decomposition is done by employing a variable purity approach such as, for instance, the SIMPLISMA (SIMPLe-to-use Interactive Self Modeling Analysis) algorithm [39,40]. This method determines the most variant samples among the collected measurements data set to be employed as the first estimation of pure spectra or pure concentration profiles. Furthermore, the analyst can specify an arbitrary variability (15% here) on the estimated spectra and concentration profiles. After this step, ALS algorithms are exploited to optimize the model and diminish the residual error, setting an arbitrary convergence criterion and number of iterations as well. In the present study, the following non-negativity (non-negative least squares algorithm [41]) and concentration closure (equal to 5 mmolL^−1^, i.e., the concentration of oxovanadium(IV), supposed to be the limiting reactant) constraints were employed for the calculation of the pure concentration profiles. Conversely, non-negativity constraint was also used for the estimation of the pure spectra of the components detected within the UV-visible spectra, while no constraints were taken into account for the assessment of the components of the recorded EPR spectra. For this purpose, the routines and the graphical user interface (GUI) developed by Jaumot et al. [38] were used on MATLAB software version R2018b.

## 5. Conclusions

The four ligands examined in this work are able to coordinate the oxovanadium(IV) cation through the bidentate kojate units, thus forming stable species in the 4–8 pH range. The shifts of the UV-vis absorption bands, as well as of the magnetic parameters (i.e., higher *g* values and lower hyperfine coupling constants) of the pentaaquo vanadyl ion [VO(H_2_O)_5_]^+2^ in the presence of the S3, S4, and SC receptors, reveal that the four ligand oxygen atoms replace the water molecules in the equatorial coordination plane, leading to the formation of the dominant species in the 4–8 pH range. In the case of S2, it could be reasonable to exclude the insertion of the second ka unit in the coordination sphere of the cation. Hydrolyzed species become important after pH 6.5, particularly for the systems with S2 and SC.

The sequestering capability of the ligands S3, S4, and SC towards oxovanadium(IV) is very similar and it is higher than that of maltol and ka.

This study paves the way to further investigations on the properties of these VO^+2^–ligand systems also in the presence of other metal cations naturally existing in physiological fluids. Moreover, additional studies to assess, both in vitro and in vivo, the toxicity, the bioavailability, and the pharmacokinetics of these species might be advisable to envisage clinical applications such as, for example, their use as insulin-mimetic compounds.

## Figures and Tables

**Table 1:** Formation constants. Protonation constants [12,13] of the ligands and formation constants for the species of oxovanadium(IV). The formation constants are obtained from the analysis of pH-metric data (ionic strength: 0.1 mol L^−1^ in KCl; temperature: 25 °C).

Ligand	S2 ^a^	S3 ^a^	S4 ^a^	SC ^a^	
**log*K* for the reaction H^+^ + LH_r_^z^ ⇆ H_r+1_L^z+1 b^**					
**Species**				**Species**	
[LH]^−^	9.85	10.20	10.59	[LH]^2−^	10.18
[LH_2_]^0^	8.10 *	8.87	9.56	[LH_2_]^−^	9.65
[LH_3_]^+^	7.56 *	7.94 *	8.02 *	[LH_3_]^0^	8.72
[LH_4_]^2+^	6.89	7.27 *	7.38 *	[LH_4_]^+^	8.04*
				[LH_5_]^2+^	7.70*
				[LH_6_]^3+^	7.10*
**log*β* ± std dev ^c^ for reaction pVO^2+^ + qL^z−^ + rH^+^ ⇆ (VO)_p_L_q_H_r_^2p+r−qz^**					
**Species**				**Species**	
[VOLH_3_]^3+^	31.64 ± 0.01	35.03 ± 0.08	36.6 ± 0.1	[VOLH_5_]^4+^	51.5 ± 0.08
[VOLH_2_]^2+^	24.57 ± 0.02	31.89 ± 0.08	33.20 ± 0.08	[VOLH_4_]^3+^	48.28 ± 0.03
[VOLH]^+^	-	22.7 ± 0.1	23.5 ± 0.1	[VOLH_3_]^2+^	40.8 ± 0.08
**log*K* for the reaction VO^2+^ + H_r_L^z^ ⇆ (VO)LH_r_^2−z^**					
**Species**				**Species**	
[VOLH_3_]^3+^	6.13	8.02	8.43	[VOLH_5_]^4+^	7.21
[VOLH_2_]^2+^	6.62	12.82	13.05	[VOLH_4_]^3+^	11.69
[VOLH]^+^	-	12.50	12.91	[VOLH_3_]^2+^	12.25
		**log*K***	**Experimental Conditions**		**Reference**
VO^2+^ + ka^−^ ⇆ VOka^+^		7.63	*I* = 0.2 mol L^−1^ KCl *T* = 25 °C		[18]
		7.61			[9]
VOka^+^ + ka^−^ ⇆ VO(ka)_2_		6.74	*I* = 0.2 mol L^−1^ KCl *T =* 25 °C		[18]
		6.89			[9]

^a^ S2: [ethane-1,2-diylbis(iminomethanediyl)]bis(5-hydroxy-4H-pyran-4-one); S3: [propane-1,3-diylbis(iminomethanediyl)]bis(5-hydroxy-4H-pyran-4-one); S4: [butane-1,4-diylbis(iminomethanediyl)]bis(5-hydroxy-4H-pyran-4-one); SC: 6,6′,6″-(((nitrilotris(ethane-2,1-diyl))tris(azanediyl))tris(methylene))tris(3-hydroxy-4H-pyran-4-one). ^b^ The protonation constants were from refs. [12,13]. The symbol * indicates the protonation of ka unit. ^c^ ±standard deviation [17].

**Table 2:** Spectrophotometric parameters. Values of λ_max_ (nm) and ε_max_ (mol^−1^ L cm^−1^) of the species in solution.

Species ^a^	Donor Set	Absorption Band						References
		Band I		Band II		Band III ^b^		
		λ_max_	ε_max_	λ_max_	ε_max_	λ_max_	ε_max_	
[VO(H_2_O)_5_]^2+^		765	16	635	7			[21]
VO(ka)_2_	2 × (CO, O^−^)	850	28	625	13	425	65	[18]
[VO(ka)_2_OH]^−^	2 × (CO, O^−^), OH^−^	870	27	595	20	450sh		
[VO(malt)]^+^	(CO, O^−^)	815	21	615	9	440	36	[20]
VO(malt)_2_	2 × (CO, O^−^)	860	27	620	12	440	73	
[VO(malt)_2_(OH)]^−^	2 × (CO, O^−^), OH^−^	870	20	605	12	450	76	
VO(HL-Mim)_2_	2 × (CO, O^−^)	860	24	620	16	540	11	
[VO(L-Mim)_2_]^2−^	2 × (CO, O^−^)	880	18	615	15	535	11	
[VO(S2)H_3_]^3+^	(CO, O^−^)	851	34.3 ± 0.1	~612 sh ^c^		~440		This work
[VO(S2)H_2_]^2+^	(CO, O^−^), OH^−^	886	35.5 ± 0.3	612	19.1 ± 0.3	~455 sh		
[VO(S3)H_3_]^3+^	(CO, O^−^)	817	28.7 ± 0.4	~613 sh		~452 sh		This work
[VO(S3)H_2_]^2+^	2 × (CO, O^−^)	860	33.8 ± 0.2	622	16.8 ± 0.3	~455 sh		
[VO(S3)H]	2 × (CO, O^−^), OH^−^	897	35 ± 1	592	21 ± 2	~469		
[VO(S4)H_3_]^3+^	(CO, O^−^)	819	28.6 ± 0.5	630	15.9 ± 0.6	-		This work
[VO(S4)H_2_]^2+^	2 × (CO, O^−^)	861	33.1 ± 0.2	619	17.5 ± 0.3	~455 sh		
[VO(S4)H]	2 × (CO, O^−^), OH^−^	898	29 ± 2	~615 sh		~480 sh		
[VO(SC)H_5_]^4+^	(CO, O^−^)	846	35.2 ± 09	612 sh		~455 sh		This work
[VO(SC)H_4_]^3+^	2 × (CO, O^−^)	854	32.0 ± 0.4	619	16.7 ± 0.4	~455 sh		
[VO(SC)H_3_]^2+^	2 × (CO, O^−^), OH^−^	884	34.8 ± 0.5	606 sh		~480 sh		

^a^ S2: [ethane-1,2-diylbis(iminomethanediyl)]bis(5-hydroxy-4H-pyran-4-one); S3: [propane-1,3-diylbis(iminomethanediyl)]bis(5-hydroxy-4H-pyran-4-one); S4: [butane-1,4-diylbis(iminomethanediyl)]bis(5-hydroxy-4H-pyran-4-one); SC: 6,6′,6″-(((nitrilotris(ethane-2,1-diyl))tris(azanediyl))tris(methylene))tris(3-hydroxy-4H-pyran-4-one). ^b^ The position of band III was estimated from the experimental spectra. ^c^ sh = shoulder.

**Table 3:** RT EPR data. Isotropic hyperfine coupling constant (*A*_0_) and isotropic *g* values (*g*_0_) of the species obtained by the analysis of EPR spectra recorded on the different oxovanadium(IV)–ligand system, as a function of pH. Errors in *g* and *A* values were estimated to be ±0.002 and ±0.5 × 10^−4^ cm^−1^, respectively.

Ligand ^a^	pH	SIM32			MCR–ALS		
		*g*_0_	*A*_0_ (cm^−1^ ∙ 10^−4^)	R ^b^	*g*_0_	*A*_0_ (cm^−1^ ∙ 10^−4^)	R ^b^
H_2_O	-	1.965	106.9	0.993	1.970	105.9	0.990
		**VO(H_2_O)_5_; VOLH_n and n−1_**			**VO(H_2_O)_5_; VOLH_n_; VOLH_n−1_**		
S2	2.5	1.964; 1968	104.6; 96.9	0.989	1.970; 1.975; 1.966	105.9; 97.7; 90.5	0.986
	3.3	1.964; 1.967	104.3; 96.0	0.985			0.984
	6.5	1.967	95.8	0.989			0.992
	7.6	1.967	94.4	0.985			0.989
	8.1	1.967	93.7	0.983			0.991
S3	2.5	1.965; 1.968	104.8; 96.8	0.985	1.970; 1.975; 1.975	105.9; 96.5; 93.2	0.986
	3.3	1.967	96.9	0.989			0.987
	6.4	1.967	95.9	0.990			0.991
	7.4	1.967	94.9	0.970			0.986
	8.0	1.967	94.0	0.981			0.988
S4	2.5	1.964; 1.968	104.4; 96.7	0.990	1.970; 1.974; 1.976	105.9; 96.1; 92.9	0.988
	3.3	1.967	96.8	0.992			0.986
	6.6	1.967	95.8	0.989			0.989
	7.4	1.967	94.9	0.983			0.986
	8.1	1.967	93.8	0.985			0.984
SC		**VO(H_2_O)_5_; VO(SC)H_5 and 4_**			**VO(H_2_O)_5_; VO(SC)H_5_; VO(SC)H_4 or 3_**		
	2.8	1.967; 1.966	104.2; 97.6	0.990	1.970; 1.974; 1.976	105.9; 96.7; 92.7	0.985
	3.1	1.967	96.9	0.994			0.991
	6.5	1.967	95.6	0.989			0.992
	7.5	1.967	94.3	0.980			0.987
	8.0	1.967	93.4	0.980			0.983

^a^ S2: [ethane-1,2-diylbis(iminomethanediyl)]bis(5-hydroxy-4H-pyran-4-one); S3: [propane-1,3-diylbis(iminomethanediyl)]bis(5-hydroxy-4H-pyran-4-one); S4: [butane-1,4-diylbis(iminomethanediyl)]bis(5-hydroxy-4H-pyran-4-one); SC: 6,6′,6″-(((nitrilotris(ethane-2,1-diyl))tris(azanediyl))tris(methylene))tris(3-hydroxy-4H-pyran-4-one). ^b^ R = Pearson’s correlation coefficient.

**Table 4:** LT EPR data. Anisotropic magnetic parameters obtained by fitting the experimental frozen solution spectra of the oxovanadium(IV) species with ka derivatives at different pH values. Anisotropic magnetic parameters for the oxovanadium(IV) cation in water at pH 3 are also shown for comparison. Errors in *g* and *A* values were estimated to be ±0.002 and ±0.5 × 10^−4^ cm^−1^, respectively.

Ligand	pH	*g*_‖_	*A*_‖_ (cm^−1^ ∙ 10^−4^)	*g*_⊥_	*A*_⊥_ (cm^−1^ ∙ 10^−4^)
H_2_O	3.0	1.933	180.8	1.978	70.2
S2	6.2	1.939	168.9	1.975	59.8
	8.2	1.941	167.2	1.974	57.1
S3	6.4	1.936	169.9	1.975	59.9
	8.2	1.939	167.1	1.972	56.3
S4	6.4	1.936	170.8	1.975	60.8
	8.0	1.940	166.9	1.973	56.9
SC	5.6	1.938	170.2	1.974	59.2
	8.0	1.938	167.0	1.971	56.8

## Supplementary Materials

The following are available online, Figures S1–S8: pH-metric titration curves, Figures S9–S11: Experimental UV-vis absorption spectra, Figures S12–S15: UV-vis absorption spectra of the chemical species, Table S1: Formation constants for the species of oxovanadium(IV) derived from the analysis of spectrophotometric data Figure S16: RT-EPR spectra, Figures S17–S18: MCR-ALS results relative to the EPR spectra, Figure S19: LT-EPR spectra.
