# Supplementary material for: Oxovanadium(IV) Coordination Compounds with Kojic Acid Derivatives in Aqueous Solution
Source: Molecules. 2019 Oct 19;24(20):3768. doi: 10.3390/molecules24203768 (PMC6833010; doi:10.3390/molecules24203768)
Supplement: Supplementary file 1 [file molecules-24-03768-s001.pdf]

Supplementary Material

Oxovanadium(IV) coordination compounds with kojic acid derivatives in aqueous solution

Silvia Berto*[1], Eugenio Alladio[1], Pier Giuseppe Daniele[1], Enzo Laurenti[1], Andrea Bono[1a], Carmelo Sgarlata[2], Gabriele Valora[2], Rosita Cappai[3], Joanna Izabela Lachowicz[4], Valeria M. Nurchi[3].

[1] *Dipartimento di Chimica, Università di Torino, Via Pietro Giuria 7, 10125 Torino, Italy; eugenio.alladio@unito.it (E.A.); piergiuseppe.daniele@unito.it (P.G.D.); enzo.laurenti@unito.it (E.L.); bonoa94@gmail.com (A.B.)*

2 *Dipartimento di Scienze Chimiche, Università degli Studi di Catania, Viale Andrea Doria 6, 95125 Catania, Italy; sgarlata@unict.it (C.S.); gabriele.valora@unict.it (G.V.)*

3 *Dipartimento di Scienze della Vita e dell'Ambiente, Università di Cagliari, Cittadella Universitaria, 09042 Cagliari, Italy; cappai@unica.it (R.C.); nurchi@unica.it (V.M.N.)*

4 *Dipartimento di Scienze Mediche e Sanità Pubblica, Università di Cagliari, Cittadella Universitaria, 09042 Monserrato, Italy; lachowicz@unica.it* (J.I.L.)

*Correspondence: silvia.berto@unito.it; phone: 0039 011 6705279

[a] Present address: Dipartimento di Chimica, Materiali e Ingegneria Chimica "Giulio Natta", Politecnico di Milano, via Mancinelli 7, 20131 Milano, Italy.

Potentiometric results

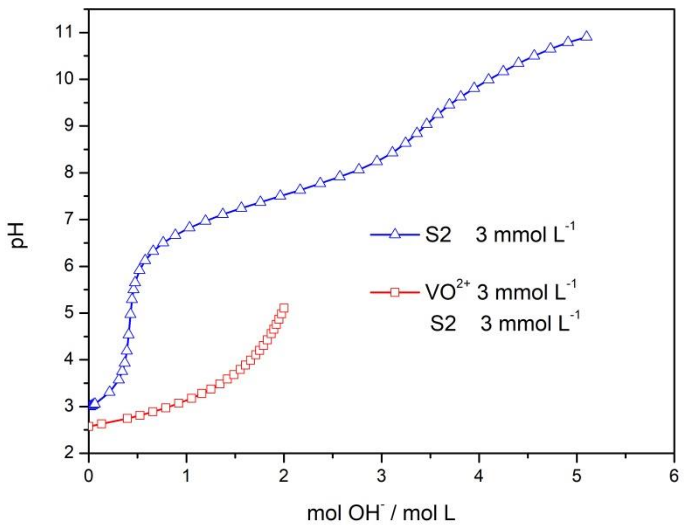

Figure S1 Titration curves recorded on solutions with S2 [ethane-1,2-diylbis(iminomethanediyl)]bis(5-hydroxy-4H-pyran-4-one)) or oxovanadium(IV) 3 mmolL^{-1} and S2 3 mmolL^{-1}, ionic strength 0.1 mol L^{-1} (KCl) and at 25°C. The equivalents of base per mole of ligand were reported on the x-axis. HCl 6 mmol L^{-1} was added to both solutions.

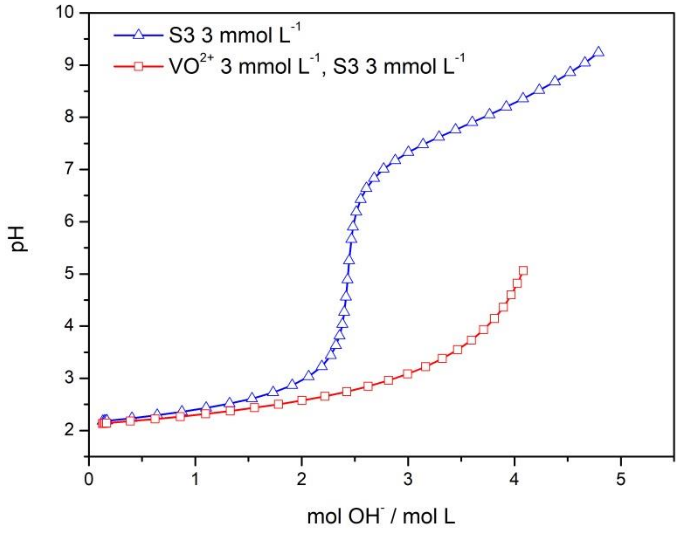

Figure S2 Titration curves recorded on solutions with S3 ([propane-1,3-diylbis(iminomethanediyl)]bis(5-hydroxy-4H-pyran-4-one)) or oxovanadium(IV) 3 mmolL^{-1} and S3 3 mmolL^{-1}, ionic strength 0.1 mol L^{-1} (KCl) and at 25°C. The equivalents of base per mole of ligand were reported on the x-axis. HCl 12 mmol L^{-1} was added to both solutions.

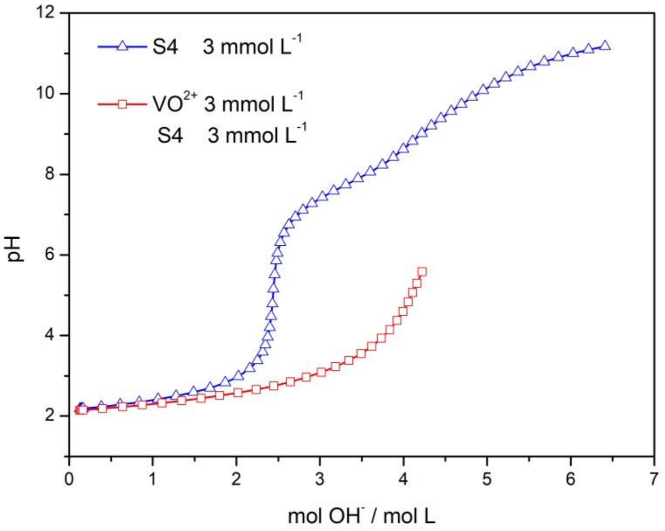

Figure S3 Titration curves recorded on solutions with S4 [butane-1,4-diylbis(iminomethanediyl)]bis(5-hydroxy-4H-pyran-4-one) or oxovanadium(IV) 3 mmolL^{-1} and S4 3 mmolL^{-1}, ionic strength 0.1 mol L^{-1} (KCl) and at 25°C. The equivalents of base per mole of ligand were reported on the x-axis. HCl 12 mmol L^{-1} was added to both solutions.

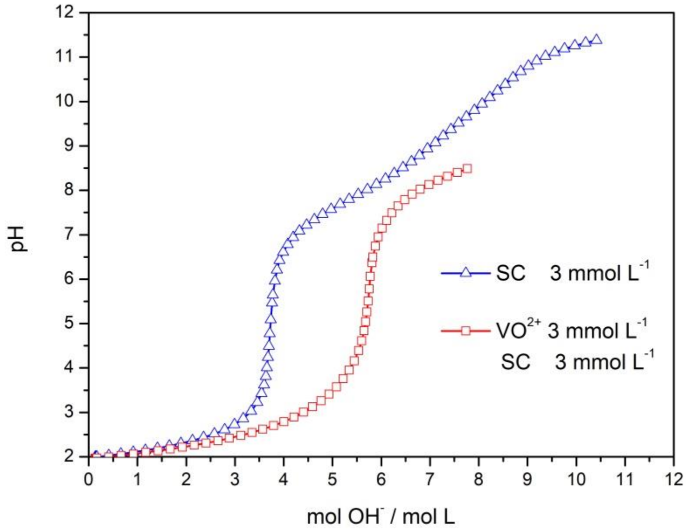

Figure S4 Titration curves recorded on solutions with SC 6,6′,6″-(((nitrilotris(ethane-2,1-diyl))tris(azanediyl))tris(methylene))tris(3-hydroxy-4H-pyran-4-one) or oxovanadium(IV) 3 mmolL^{-1} and SC 3 mmolL^{-1}, ionic strength 0.1 mol L^{-1} (KCl) and at 25°C. The equivalents of base per mole of ligand were reported on the x-axis. HCl 18 mmol L^{-1} was added to both solutions.

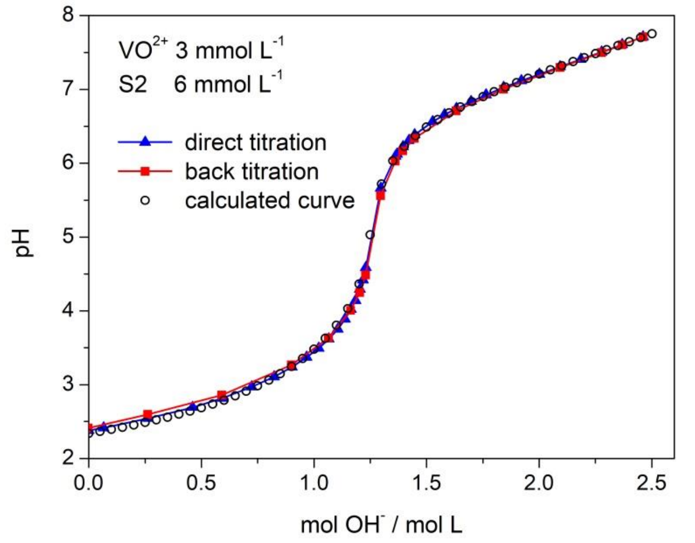

Figure S5 Direct and back titration curves, recorded manually on the system oxovanadium(IV)-S2 (HCl 12 mmol L^{-1} was added), and calculated curve (formation constants reported in the Table 1 of the main text); oxovanadium(IV) 3 $mmolL^{-1}$, S2 6 $mmolL^{-1}$, HCl 12 mmol L^{-1}, ionic strength 0.1 mol L^{-1} (KCl) and at 25°C. The equivalents of base per mole of ligand were reported on the x-axis.

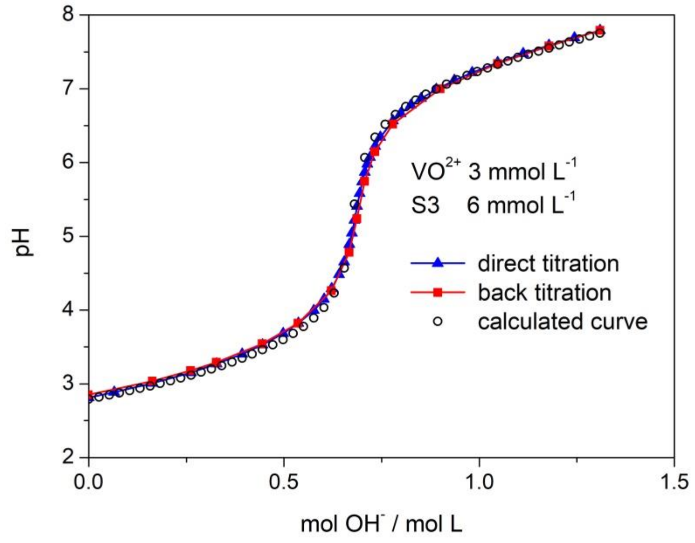

Figure S6 Direct and back titration curves, recorded manually on the system oxovanadium(IV)-S3, and calculated curve (formation constants reported in the Table 1 of the main text); oxovanadium(IV) 3 $mmolL^{-1}$, S3 6 $mmolL^{-1}$, HCl 8 mmol L^{-1}, ionic strength 0.1 mol L^{-1} (KCl) and at 25°C. The equivalents of base per mole of ligand were reported on the x-axis.

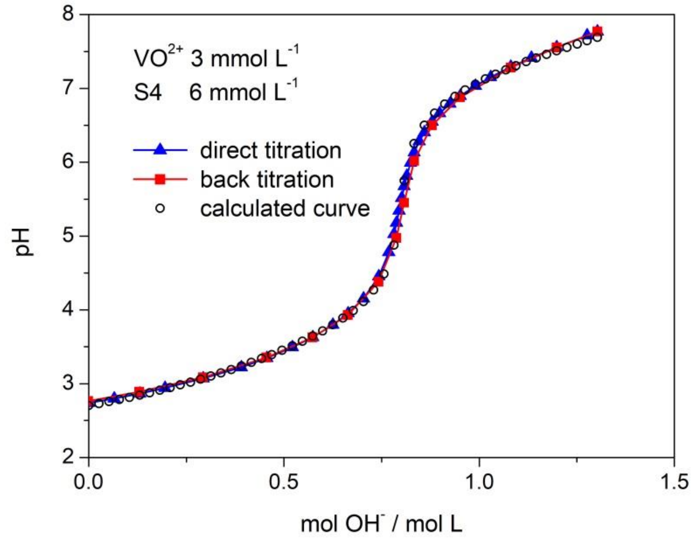

Figure S7 Direct and back titration curves, recorded manually on the system oxovanadium(IV)-S4, and calculated curve (formation constants reported in the Table 1 of the main text); oxovanadium(IV) 3 mmolL^{-1}, S4 6 mmolL^{-1}, HCl 8 mmol L^{-1}, ionic strength 0.1 mol L^{-1} (KCl), and at 25°C. The equivalents of base per mole of ligand were reported on the x-axis.

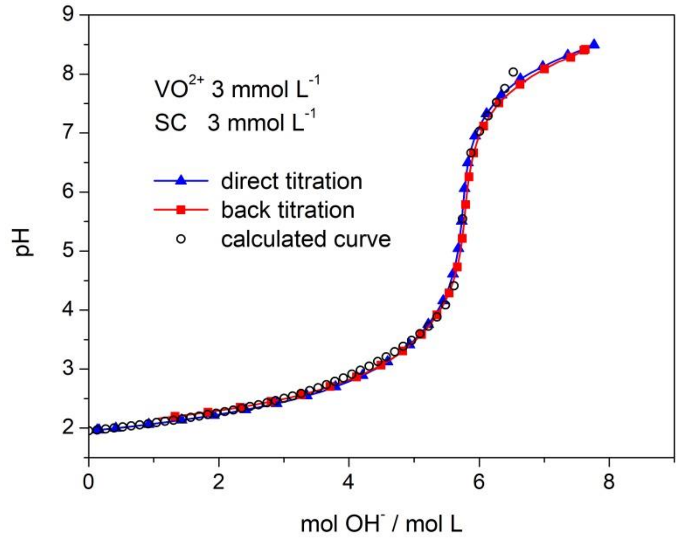

Figure S8 Direct and back titration curves, recorded manually on the system oxovanadium(IV)-SC, and calculated curve (formation constants reported in the Table 1 of the main text); oxovanadium(IV) 3 mmolL^{-1}, SC 3 mmolL^{-1}, HCl 18 mmol L^{-1}, ionic strength 0.1 mol L^{-1} (KCl) and at 25°C. The equivalents of base per mole of ligand were reported on the x-axis.

Spectrophotometric results

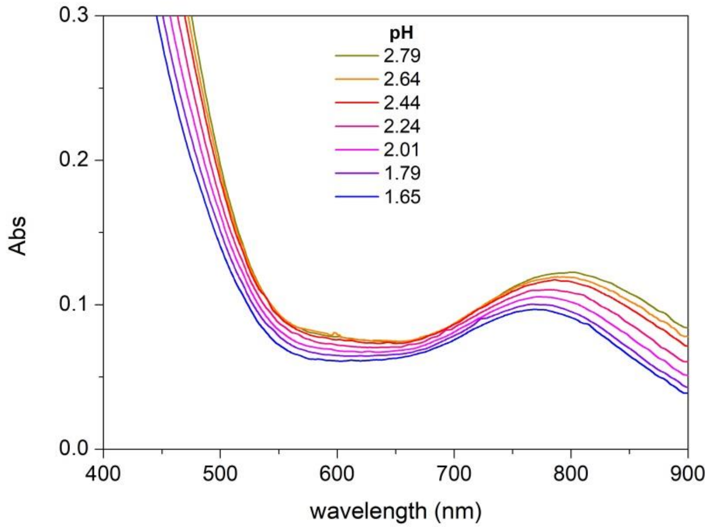

Figure S9 UV-vis absorption spectra of solutions containing oxovanadium(IV) 5 mmolL^{-1} and S3 5 mmolL^{-1} (batch titration).

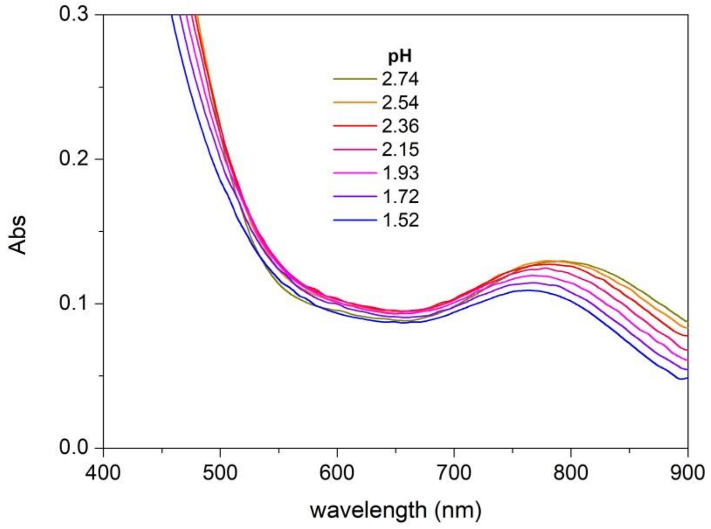

Figure S10 UV-vis absorption spectra of solutions containing oxovanadium(IV) 5 mmolL^{-1} and S4 5 mmolL^{-1} (batch titration).

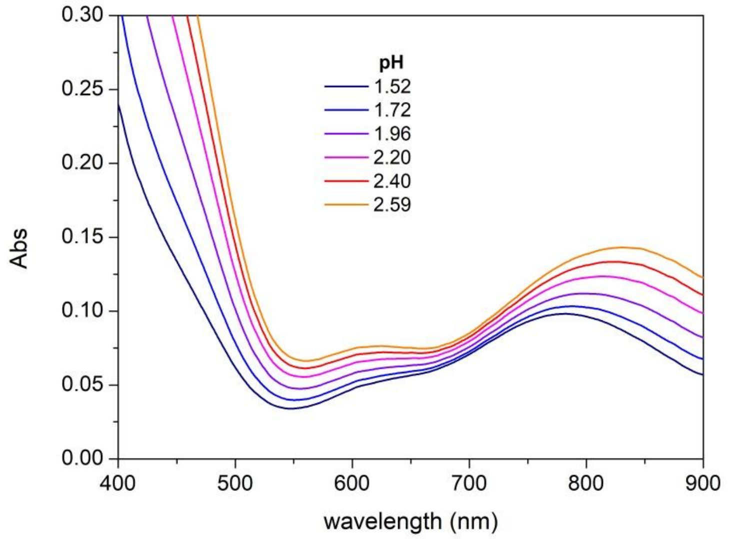

Figure S11 UV-vis absorption spectra of solutions containing oxovanadium(IV) 5 mmolL^{-1} and SC 5 mmolL^{-1} (batch titration).

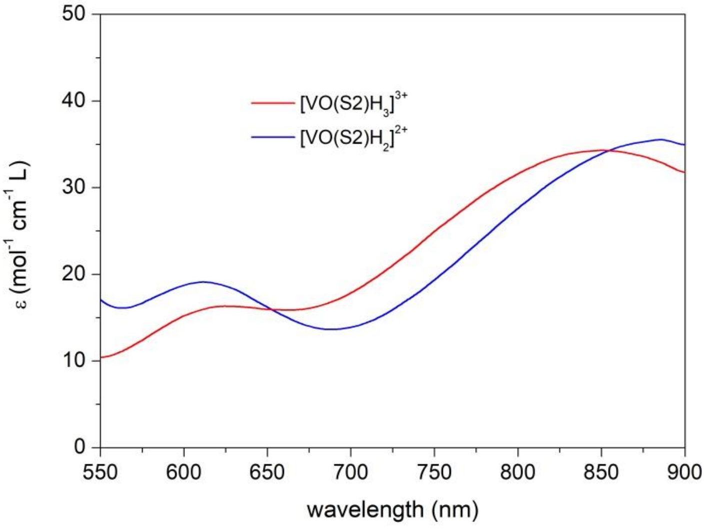

Figure S12 Absorption spectra of the complexes of oxovanadium(IV) with S2.

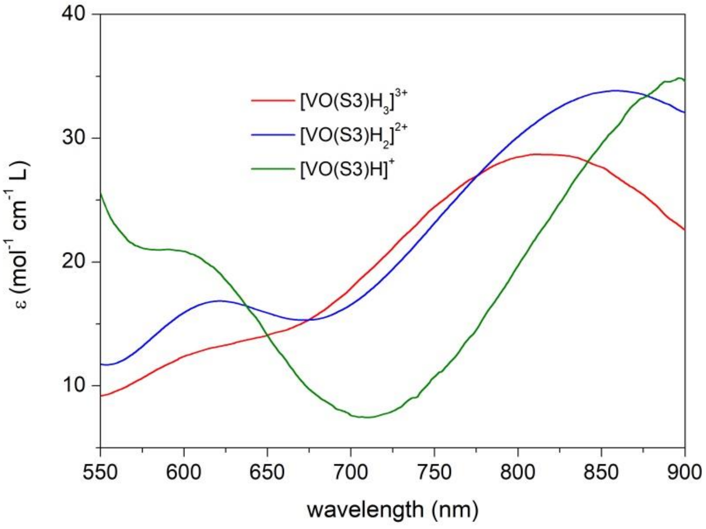

Figure S13 Absorption spectra of the complexes of oxovanadium(IV) with S3.

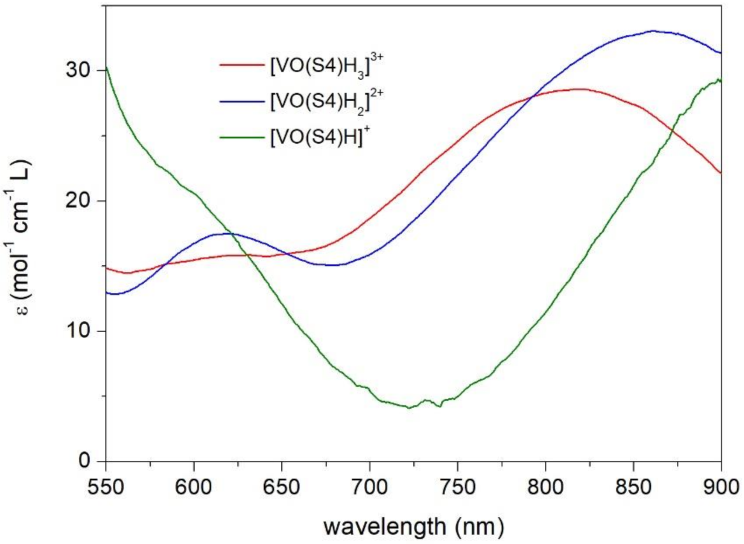

Figure S14 Absorption spectra of the complexes of oxovanadium(IV) with S4.

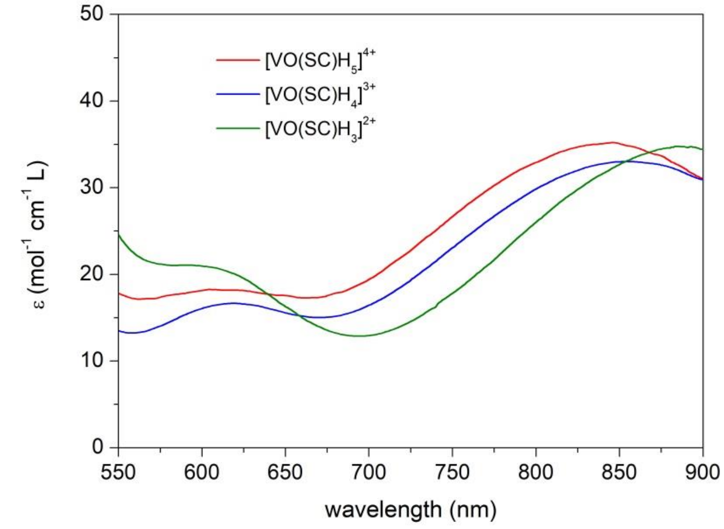

Figure S15 Calculated absorption spectra of the complexes of oxovanadium(IV) with SC.

Table S1 Formation constants for the species of oxovanadium(IV) derived from the analysis of spectrophotometric data (ionic strength: 0.1 mol L^{-1} in KCl; temperature: 25°C).

**Ligand**	**S2** [a]	**S3** [a]	**S4** [a]		**SC** [a]
**log*β* ± std dev** [b] **for reaction $pVO^{2+} + qL^{z-} + rH^{+} \leftrightarrows (VO)_pL_qH_r^{2p+r-qz}$**					
**Species**				**Species**	
$[VOLH_3]^{3+}$	32.15±0.01	34.7±0.02	36.3±0.02	$[VOLH_5]^{4+}$	51.21±0.03
$[VOLH_2]^{2+}$	25.07±0.02	31.2±0.03	32.8±0.04	$[VOLH_4]^{3+}$	47.96±0.04
$[VOLH]^{+}$		22.9±0.04	24.0±0.07	$[VOLH_3]^{2+}$	40.81±0.04
**log*K* for the reaction $VO^{2+} + H_rL^{z} \leftrightarrows (VO)LH_r^{2-z}$**					
**Species**				**Species**	
$[VOLH_3]^{3+}$	6.64	7.69	8.13	$[VOLH_5]^{4+}$	6.92
$[VOLH_2]^{2+}$	7.12	12.13	12.65	$[VOLH_4]^{3+}$	11.37
$[VOLH]^{+}$	-	12.70	13.41	$[VOLH_3]^{2+}$	12.26

[a] S2: [ethane-1,2-diylbis(iminomethanediyl)]bis(5-hydroxy-4H-pyran-4-one);
S3: [propane-1,3-diylbis(iminomethanediyl)]bis(5-hydroxy-4H-pyran-4-one);
S4: [butane-1,4-diylbis(iminomethanediyl)]bis(5-hydroxy-4H-pyran-4-one);
SC: 6,6′,6″-(((nitrilotris(ethane-2,1-diyl))tris(azanediyl))tris(methylene))tris(3-hydroxy-4H-pyran-4-one).

[b] ±standard deviation [14]

EPR results

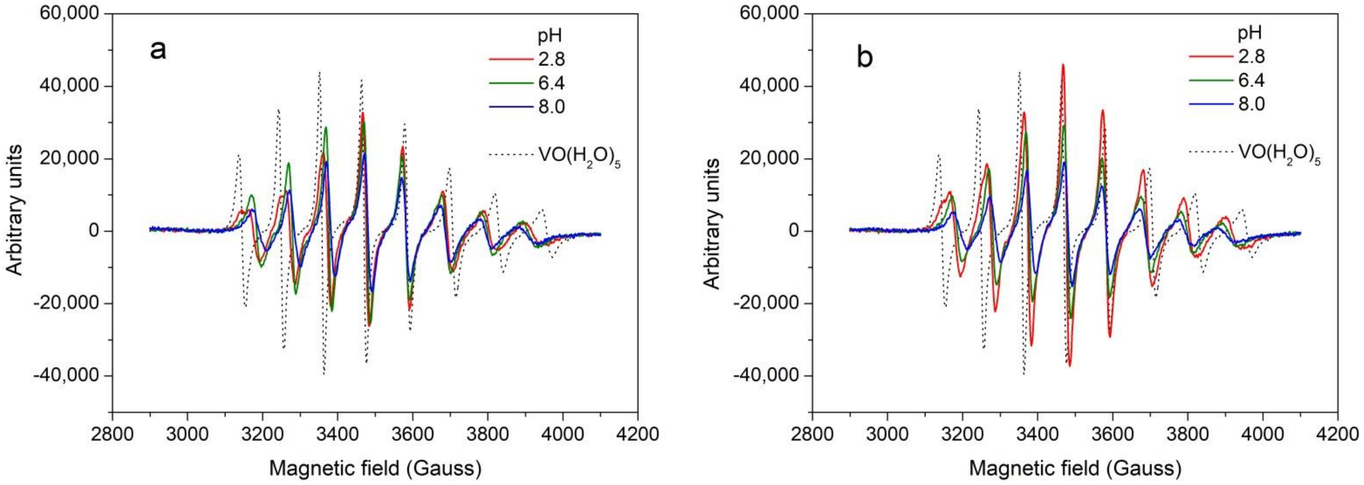

Figure S16 RT-EPR spectra of solutions with oxovanadium(IV) 5 mmolL^{-1} and S3 15 mmolL^{-1} (a); oxovanadium(IV) 5 mmolL^{-1} and SC 15 mmolL^{-1}(b).

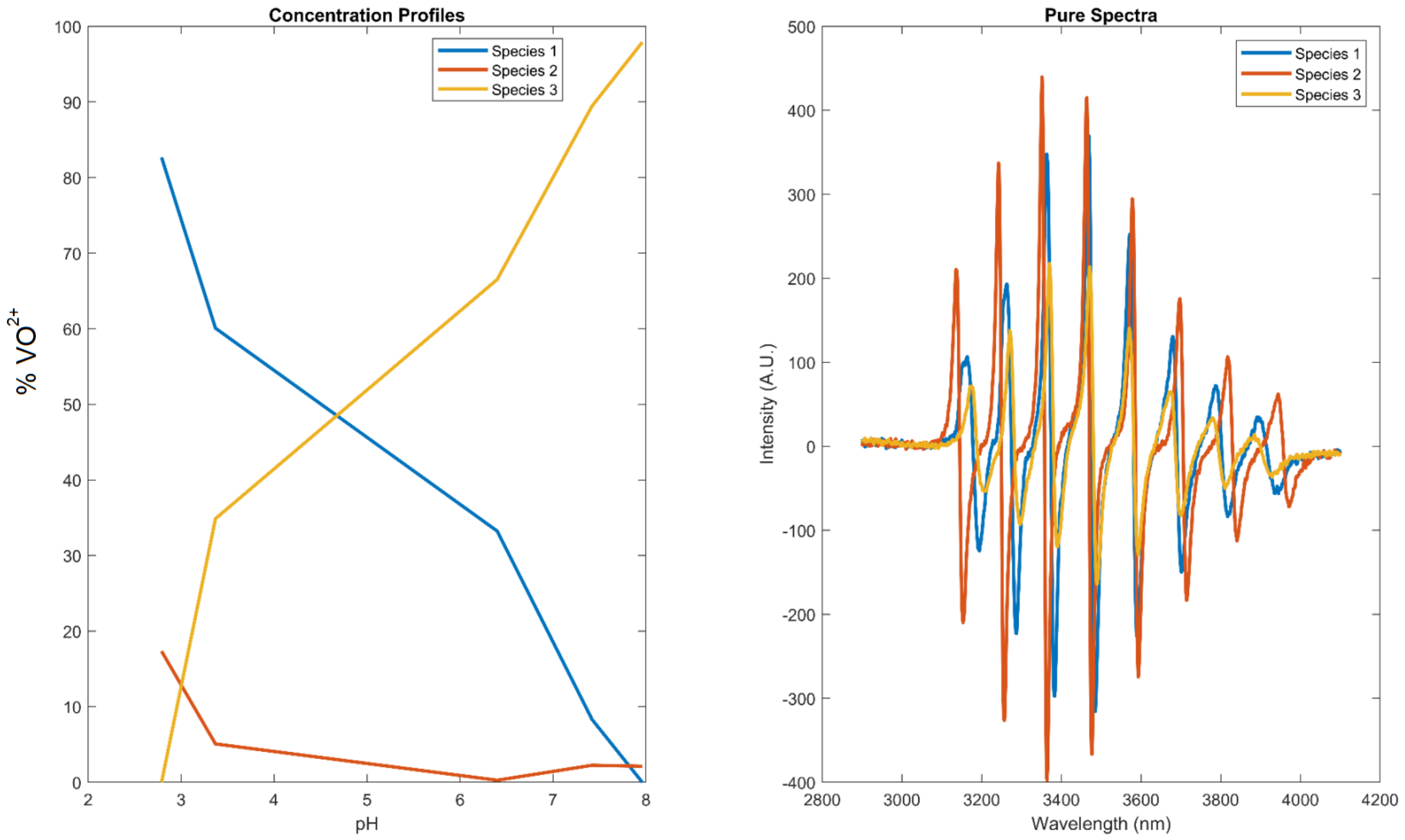

Figure S17 MCR-ALS results relative to the EPR spectra. MCR-ALS results relative to the EPR spectra of the system involving solutions with oxovanadium(IV) 5 mmolL^{-1} and S3 15 mmolL^{-1}. The pure relative concentration profiles (left) calculated by MCR-ALS correspond to the first (blue line), the second (red line) and the third (yellow line) components of the evaluated sample solutions. Their relative extrapolated pure spectra are reported (right), too. The species 1, 2 and 3 can be associated to $[VO(S3)H_3]^{3+}$, $[VO(H_2O)_5]^{2+}$ and $[VO(S3)H_2]^{2+}$, respectively.

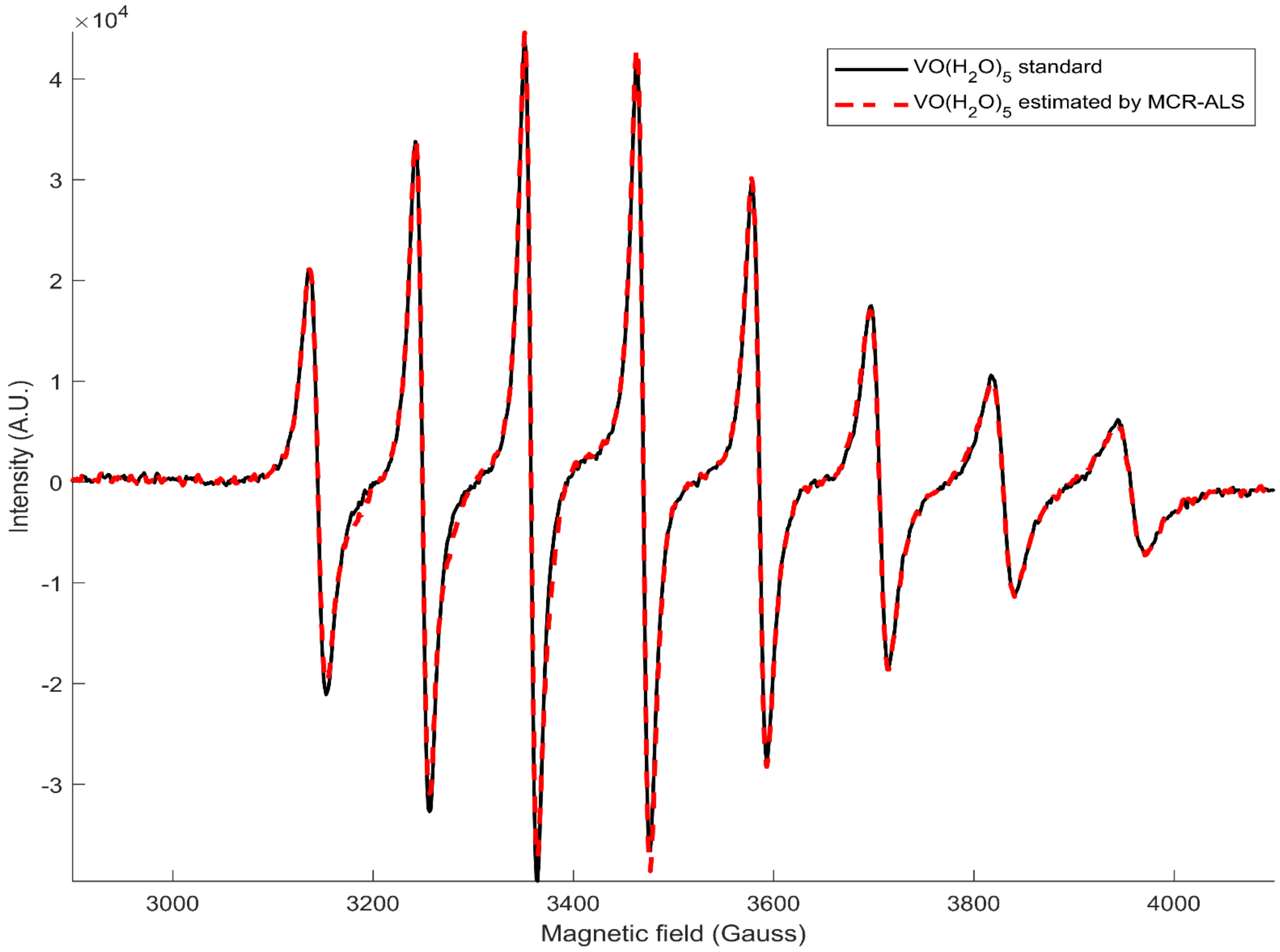

Figure S18 Experimentally recorded pure spectra of oxovanadium(IV) 5 mmolL^{-1} (solid black line) and the pure spectra calculated by MCR-ALS (dashed red line).

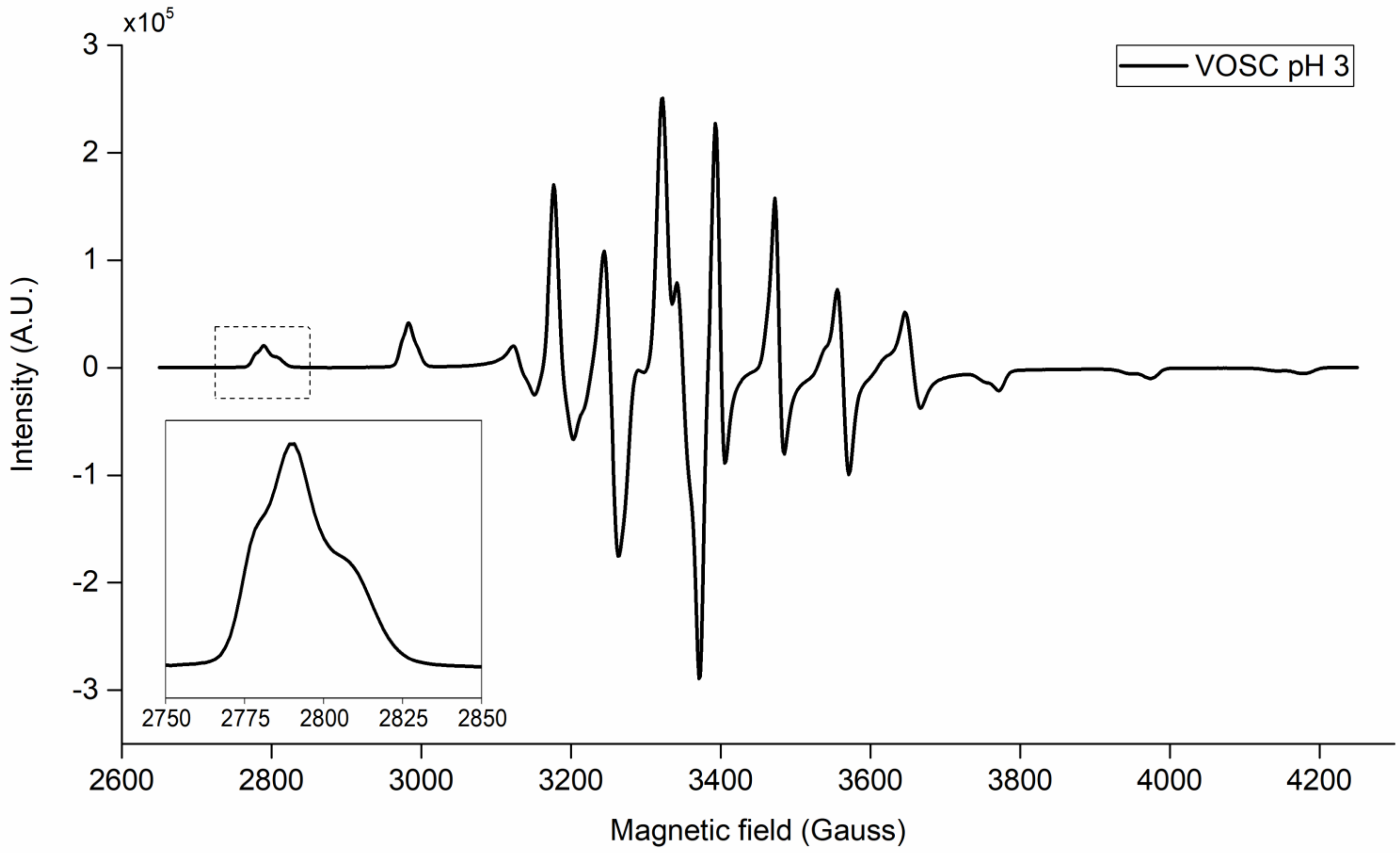

Figure S19 Experimental LT EPR spectrum of VOSC frozen solution at pH 3. The inset is an expansion of the first EPR transition (M_I = -7/2) in the low field parallel region that shows more than one species contributing to the overall EPR spectrum.
